# Differences in clinical characteristics and treatment outcomes of submacular hemorrhage caused by age-related macular degeneration and retinal macroaneurysms: A multicenter survey from the Japan Clinical Retina Study (J-CREST) group

**DOI:** 10.1371/journal.pone.0274508

**Published:** 2022-09-29

**Authors:** Takeshi Kimura, Takashi Araki, Tsutomu Yasukawa, Aki Kato, Soichiro Kuwayama, Takamasa Kinoshita, Fumiki Okamoto, Tomoya Murakami, Yoshinori Mitamura, Taiji Sakamoto, Hiroto Terasaki, Sentaro Kusuhara, Akiko Miki, Yoshihiro Takamura, Mineo Kondo, Hisashi Matsubara, Tetsuo Ueda, Hiroki Tsujinaka, Fumi Gomi

**Affiliations:** 1 Department of Ophthalmology, Hyogo College of Medicine, Nishinomiya, Japan; 2 Department of Ophthalmology and Visual Science, Nagoya City University Graduate School of Medical Sciences, Nagoya, Japan; 3 Department of Ophthalmology, Sapporo City General Hospital, Sapporo, Japan; 4 Department of Ophthalmology, Faculty of Medicine, University of Tsukuba, Tsukuba, Japan; 5 Department of Ophthalmology, Tokushima University Graduate School, Tokushima, Japan; 6 Department of Ophthalmology, Kagoshima University Graduate School of Medical and Dental Sciences, Kagoshima, Japan; 7 Department of Surgery, Division of Ophthalmology, Kobe University Graduate School of Medicine, Kobe, Japan; 8 Department of Ophthalmology, Fukui University Graduate School of Medical Sciences, Yoshida, Japan; 9 Department of Ophthalmology, Mie University Graduate School of Medicine, Tsu, Japan; 10 Department of Ophthalmology, Nara Medical University, Kashihara, Japan; National Yang-Ming University Hospital, TAIWAN

## Abstract

**Purpose:**

To evaluate the clinical characteristics, treatment trends, and visual prognosis of submacular hemorrhage (SMH) secondary to neovascular age-related macular degeneration (nAMD) and retinal arterial macroaneurysm (RAM).

**Methods:**

This retrospective study enrolled 187 Japanese patients with SMH at 10 institutions from 2015 to 2018. Medical records including SMH etiology, best-corrected visual acuity (BCVA), fundus photographs, optical coherence tomography images, and selected treatments were analyzed.

**Results:**

Major causes of SMH were typical nAMD (tnAMD) (18%), polypoidal choroidal vasculopathy (PCV) (50%) and RAM (29%). Age, male/female ratio, baseline BCVA, central retinal thickness, and involved retinal layers were significantly different between etiologies (all P<0.0001). Treatment with anti-vascular endothelial growth factor drugs with and without intravitreal gas injection was chosen for half of eyes in the tnAMD and PCV groups, whereas vitrectomy was performed in 83.7% of eyes with RAM. The final BCVA improved significantly from baseline in the PCV and RAM groups (P = 0.0009, P<0.0001) and final BCVA was significantly better in the PCV group at a level similar to the other groups (P = 0.0007, P = 0.0008). BCVA improvement from baseline was significantly greater in the RAM group compared with the tnAMD (P = 0.0152) and PCV (P = 0.017) groups. Multivariate analysis revealed better final BCVA was significantly associated with younger age (P = 0.0054), better baseline BCVA (P = 0.0021), RAM subtype (P = 0.0446), and no tnAMD (P = 0.001).

**Conclusions:**

The characteristics of, and treatment strategy for, SMH were different between the underlying diseases. Anti-vascular endothelial growth factor treatment with or without expansile gas was mainly chosen for SMH in tnAMD and PCV, whereas vitrectomy with gas was the most common treatment for RAM, and the higher rate for vitrectomy might result in the greater BCVA improvement in the RAM group than in the other groups. Final BCVA was better in PCV, RAM, and tnAMD, in that order, because patients with PCV were younger and had better baseline BCVA.

## Introduction

Submacular hemorrhage (SMH) is an accumulation of blood in the macular area. When untreated, it causes irreversible damage to photoreceptors, resulting in significant visual deterioration. Neovascular age-related macular degeneration (nAMD) and retinal arterial macroaneurysm (RAM) are two major causes of SMH [1–3]. In Asia, polypoidal choroidal vasculopathy (PCV), which is a subtype of nAMD characterized by polyp-like choroidal vascular dilation, is frequently associated with SMH [4, 5]. Polyp regression is important for the prevention of SMH, and various clinical studies have shown that anti-vascular endothelial growth factor (VEGF) therapy is effective for PCV [6, 7]. In addition, a previous study showed that photodynamic therapy combined with anti-VEGF therapy for PCV decreased the rate of development of photodynamic therapy-related hemorrhage [8]. Now that anti-VEGF drugs are widely used for the treatment of nAMD, the causes of SMH may be changing.

The goal of treatment for SMH is to remove the hemorrhage from the macula or at least from the fovea. Various treatments have been attempted, including the intravitreal injection of expansile gas with and without tissue plasminogen activator (tPA), intravitreal injection of anti-VEGF alone or combined with gas, and vitrectomy with the intravitreal or subretinal injection of tPA (and air) and/or an intravitreal gas [2, 3, 9–13]. However, there is no standardized treatment protocol for SMH because the visual prognosis after treatment is difficult to estimate because of the variation of SMH characteristics, including the cause, duration, volume, and location of hemorrhage.

SMH can be diagnosed based on the appearance of the fundus. Optical coherence tomography (OCT) enables the depiction of the detailed distribution of hemorrhage in various layers, such as the preretinal (subhyaloid or sub-internal limiting membrane), intraretinal, subretinal, and subretinal pigment epithelium (sub-RPE). A classification of SMH recently proposed by researchers from Europe termed FLATCAPS includes the etiology, location, and size of hemorrhage based on fundus photographs and OCT findings [1]. Using this classification system, we attempted to differentiate SMH in a large number of Japanese patients. We also investigated the current treatment trends and visual prognosis according to the causative disease.

## Patients and methods

This was a multicenter retrospective study and all participating institutions were part of the Japan Clinical Retina Study (J-CREST). The Institutional Review Board of Hyogo College of Medicine approved this study (No.3113). This study adhered to the tenets of the Declaration of Helsinki and was approved by the ethics committees of all participating centers (Hyogo College of Medicine, Nagoya City University Graduate School of Medical Sciences, Sapporo City General Hospital, Tsukuba University, Tokushima University, Kagoshima University, Kobe University, Fukui University, Mie University Graduate School of Medicine, and Nara Medical University. All ethics committees permitted the opt-out approach to obtain consent from the participants. The data acquired in the course of the data analysis were anonymized before we accessed them.

The study included patients with recent-onset SMH in either eye who were examined from April 2015 to September 2018. The inclusion criteria were SMH affecting more than two optic disc areas involving the fovea, a ≤1-month duration of SMH-associated visual deterioration, and a follow-up period of ≥2 months from baseline (or from the initial treatment for SMH if it was treated). The exclusion criteria were recurrent SMH and the presence of vision-affecting retinal and/or choroidal diseases unrelated to SMH.

The following medical data were collected: patient age, sex, history of anticoagulant therapy, duration of SMH, possible diagnosis for the cause of SMH, and selected treatment. The possible diagnosis was determined by the treating physicians among typical nAMD (tnAMD), PCV, RAM, and unknown. The diagnosis of PCV was determined by the presence of presumed polypoidal lesions from fundus, OCT (highly elevated RPE line or notch of pigment epithelial detachment) or indocyanine green angiography findings and previous medical records. An ophthalmological examination including best-corrected visual acuity (BCVA), color fundus photography, and OCT was performed at baseline, 1 month after baseline or after the initiation of intervention (if any), and at the final visit. The findings of SMH were assessed by color fundus photography and OCT. The central retinal thickness (CRT) was defined as the distance between the inner surface of inner limiting membrane (ILM) and the outer portion of the hyper-reflective line matching the RPE on a B-scan OCT image, and this was measured in all patients. Part of the data from subjects with SMH secondary to nAMD were included in our different study [14].

### FLATCAPS classification

The characteristics of SMH were classified using the FLATCAPS (Foveal Involvement, Retinal Layers, Age/Duration, Thickness, Cause/Pathogenesis, Size) system [1]. Because this study included eyes with SMH involving the fovea, the other items in the classification system (retinal layers, age/duration, thickness, cause, and size) were investigated. The layers involved in the hemorrhage were defined as follows: L0, preretinal layer (subhyaloidal/sub-ILM); L1, subretinal layer; L2, sub-RPE; L3, mixed subretinal layer and sub-RPE; and L4, blood present in more than two of the above-mentioned spaces. The hemorrhage thickness under the fovea was classified as follows: T0, <500 μm; T1, 500–1000 μm; and T2, >1000 μm. The extent of hemorrhage was classified as follows: S0, <1 disc area; S1, 1–5 disc areas; S2, >5 disc areas and up to the arcades; and S3, massive hemorrhage exceeding the arcades. The duration from onset to intervention was classified as follows: A0, ≤7 days; A1, 8–14 days; and A2, >14 days.

### Statistical analysis

Statistical analysis was performed with JMP^®^ Pro 14 (SAS Institute, Cary, NC, USA). The decimal BCVA was converted to the logarithm of the minimum angle of resolution (logMAR) for statistical analysis. Numerical variables are shown as the mean and standard deviation. The subgroup analysis was performed using Wilcoxon’s rank test, Kruskal-Wallis test, and Pearson’s chi-square test. Paired *t*-tests were used to compare BCVA at baseline and thereafter. We conducted a multivariate analysis to explore the factors influencing the selection of treatment and later BCVA. A P-value of <0.05 was considered statistically significant.

## Results

### Patients’ demographics

Overall, 196 eyes of 196 Japanese patients were analyzed initially, and 9 were excluded for the following reasons: not meeting the inclusion criteria (2 eyes), meeting the exclusion criteria (2 eyes), low image quality because of accompanying vitreous hemorrhage (3 eyes), and insufficient clinical data (2 eyes). Finally, 187 eyes of 187 patients were analyzed.

The cause of SMH was nAMD in 128 eyes (68%) [including typical nAMD (tnAMD) in 33 eyes (17%), PCV in 94 eyes (50%), and retinal angiomatous proliferation in 1 eye (1%)], RAM in 54 eyes (29%), and unknown in 5 eyes (3%). Table 1 shows the baseline characteristics by the etiology of SMH among the tnAMD, PCV, and RAM groups. Patient age and sex were significantly different between groups (both P < 0.0001), patients in the PCV group were younger than those in the other groups, and those in the RAM group were predominantly women. Overall, 51 patients were treated with anticoagulant therapy. The mean time from onset of SMH to the hospital visit was 10.8 ± 14.9 days and it was significantly different between the groups (P = 0.0233); it was longer in tnAMD group than the other groups. The baseline BCVA was significantly different between the groups (P < 0.0001): baseline BCVA in the PCV group was better than in the other groups. The CRT was significantly different between the groups and the RAM group had the highest thickness. Thirteen of 33 patients (39%) in the tnAMD group and 26 of 94 (28%) patients in the PCV group had one or more histories of anti-VEGF therapy.

**Table 1:** Clinical characteristics of study patients.

	tnAMD (n = 33)	PCV (n = 94)	RAM (n = 54)	P-value
Age, years	77.2 ± 8.2	73.8 ± 9.3	80.1 ± 7.8	<0.0001*
Sex, male/female	23/10	65/29	14/40	<0.0001*
Anticoagulant therapy	14 (57.6%)	22 (23.4%)	13 (24.1%)	0.1038
Duration from SMH, days	20.3 ± 27.1	9.3 ±11.0	8.67 ± 9.06	0.0233*
Lens status, phakic /pseudophakia/aphakia	20/12/1	65/28/1	37/17/0	0.6773
Baseline BCVA (log MAR)	1.06 ± 0.61	0.82 ± 0.54	1.28 ± 0.51	<0.0001*
Central retinal thickness, μm	627.5 ± 170.4	549.5 ± 237.3	811.0 ± 318.2	<0.0001*
Previous treatment	13 (39.3%)	26 (27.7%)	0 (0%)	<0.0001*

Data are presented as the mean ± standard deviation, n, or n (%).

tnAMD = typical neovascular age-related macular degeneration; PCV = polypoidal choroidal vasculopathy; RAM = retinal arterial microaneurysm; SMH = submacular hemorrhage; BCVA = best-corrected visual acuity.

*Statistically significant.

### Classification of SMH using FLATCAPS among tnAMD, PCV, and RAM

**Fig 1:**
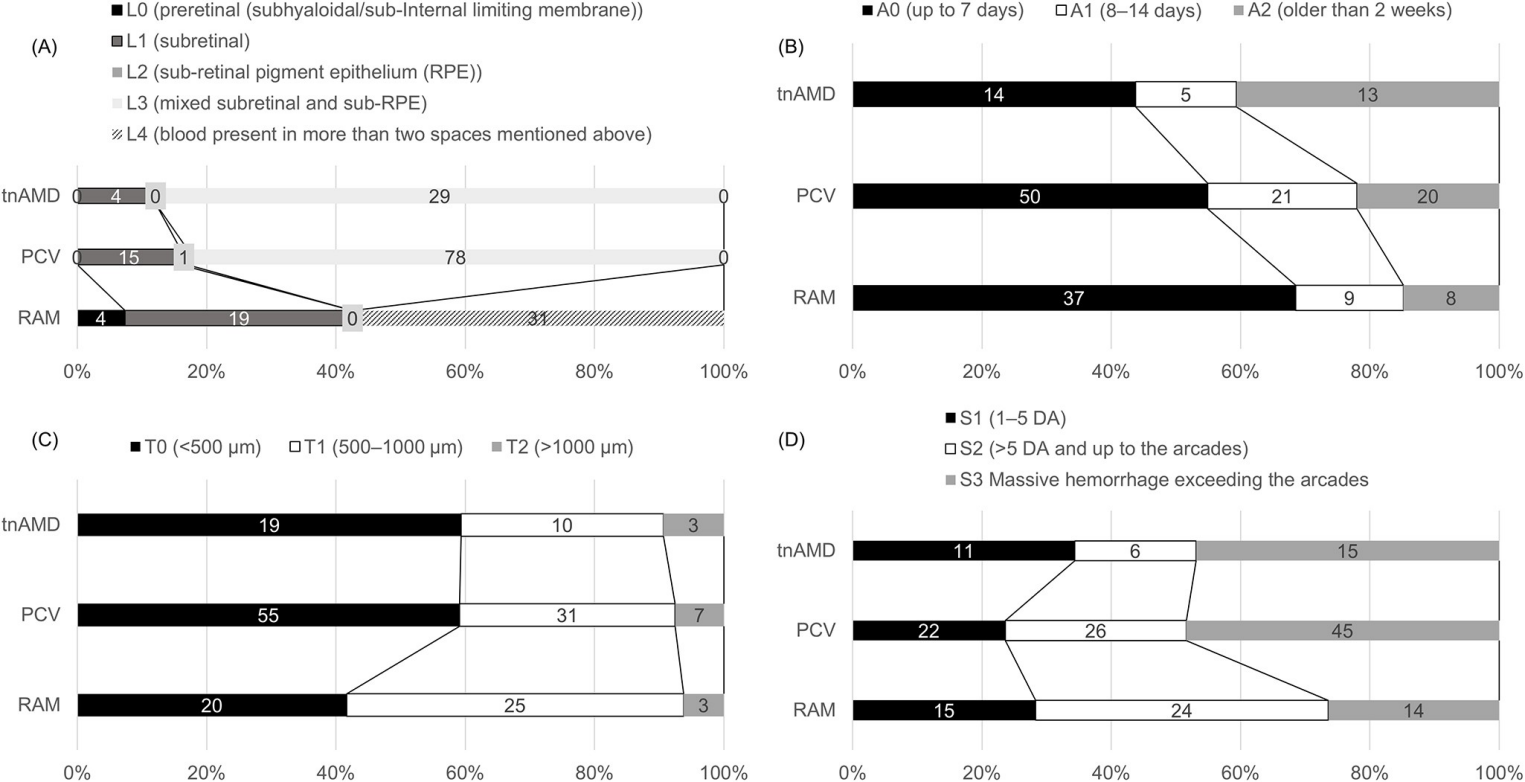
Classification of SMH in cases with tnAMD, PCV, and RAM by FLATCAPS classification. (A) Layers involving hemorrhage. (B) Duration of symptoms. (C) Thickness of hemorrhage. (D) Extent of hemorrhage. tnAMD: typical neovascular age-related macular degeneration. PCV: polypoidal choroidal vasculopathy RAM: retinal arterial microaneurysm, RPE: retinal pigment epithelium, DA: disc area.

The ratio of each item in the FLATCAPS classification according to the cause of SMH is summarized in Fig 1. Fig 1A shows the layers involved in the hemorrhage. The item with the highest proportion of eyes was L3 in the tnAMD (88%) and PCV (83%) groups, and L4 in the RAM group (60%). Fig 1B shows the duration of symptoms. The rate of eyes classified as A2 was higher in the tnAMD group than the other groups. Regarding the thickness of SMH, 60% of eyes in both the tnAMD and the PCV group were classified as T0, whereas in the RAM group, nearly 60% of eyes were T1 (Fig 1C). Regarding the size of hemorrhage, the RAM group had fewer eyes where SMH exceeded the arcades (Fig 1D).

The thickness of SMH in patients with anticoagulant therapy (T0 = 48%, T1 = 38%, T2 = 16%) was significantly greater than that in patients without anticoagulant therapy (T0 = 56%, T1 = 39%, T2 = 5%) (P = 0.0484); however, the size of hemorrhage was not significantly different between patients with and without anticoagulant therapy (S0 = 28% and 24%, S1 = 31% and 33%, and S2 = 41% and 43%, respectively) (P = 0.8151).

### Selected treatments

Overall, 173 eyes (95.1%) underwent treatment for SMH: 97.0% of eyes in the tnAMD group, 96.8% in the PCV group, and 92.6% in the RAM group. Table 2 shows the selected treatment strategies in each group. Anti-VEGF therapy with and without the intravitreal injection of expansile gas and/or tPA was administered in 50% and 43% of eyes with tnAMD and PCV, respectively and the rate of treatment with anti-VEGF therapy was significantly lower for eyes with RAM (10.2%) (P < 0.0001). Expansile gas injection was also more frequently used for tnAMD or PCV compared with RAM (P = 0.012), and it was mostly combined with intravitreal tPA injection. Pars plana vitrectomy (PPV) with gas tamponade was conducted in 30 eyes (24%) from the tnAMD and PCV groups and 37 eyes (74%) in the RAM group, with significantly more cases of PPV in the RAM group than in the other groups (P < 0.0001). Combined subretinal tPA injection was selected for 37 eyes (48%), including 6/8 (75%) in the tnAMD group, 15/22 (68%) in the PCV group, and 16/37 (43%) in the RAM group. Two eyes received subretinal gas/air injection.

**Table 2:** Initially selected treatments in eyes with SMH in tnAMD, PCV and RAM groups.

	tnAMD group (n = 32)	PCV group (n = 91)	RAM group (n = 50)	P-value
**Anti-VEGF**	16 (50.0%)	39 (42.9%)	5 (10.0%)	0.0001
Monotherapy	12 (37.5%)	24 (26.4%)	4 (8.0%)	
With expansile gas	1 (3.1%)	5 (5.5%)	1 (2.0%)	
With t-PA	0 (0%)	2 (2.2%)	0 (0.0%)	
With t-PA and expansile gas	3 (9.4%)	8 (8.8%)	0 (0.0%)	
**Expansile gas**	8 (25.0%)	24 (26.4%)	3 (6.0%)	0.012
Monotherapy	2 (6.3%)	6 (6.6%)	1 (2.0%)	
With tPA	6 (18.8%)	18 (19.8%)	2 (4.0%)	
**PPV with expansile gas**	8 (25.0%)	22 (24.2%)	37 (74.0%)	< 0.0001
With anti-VEGF	1 (3.1%)	15 (16.5%)	1 (2.0%)	
With intravitreal t-PA	1 (3.1%)	2 (2.2%)	6 (12.0%)	
With subretinal t-PA	6 (18.8%)	15 (16.5%)	16 (32.0%)	

Data are presented as n (%).

SMH = submacular hemorrhage; tnAMD = typical neovascular age-related macular degeneration; PCV = polypoidal choroidal vasculopathy; RAM = retinal arterial microaneurysm; VEGF = vascular endothelial growth factor; t-PA = tissue plasminogen activator; PPV = pars plana vitrectomy.

*Statistically significant.

Logistic regression analysis showed that significant factors associated with PPV were better baseline BCVA (P = 0.0001), diagnosis of RAM (P < 0.0001), no diagnosis of tnAMD (P = 0.0063), and duration of SMH within 1 week (P = 0.0155). The size of SMH was a possible factor (P = 0.0631) associated with PPV, but age, SMH thickness, and involved layers were not.

### Time course

The median follow-up period in each group was 18, 14.5, and 6 months in the tnAMD, PCV, and RAM groups (Table 3), which was significantly different between the groups (P < 0.0001). The RAM group had a shorter median follow-up period than the other groups (P = 0.0004 [tnAMD group], P < 0.0001 [PCV group]). Of 24 eyes (73%) in the tnAMD group, 78 eyes (83%) in the PCV group, and 7 eyes (13%) in the RAM group, a mean of 5.5, 3.9, and 1.4 injections of anti-VEGF drug were conducted after the initial treatment. Photocoagulation was performed in one eye with RAM.

**Table 3:** The course of best-corrected visual acuity among the tnAMD, PCV, and RAM groups.

	tnAMD	PCV	RAM	P-value
Median months of follow-up time (range)	13 (3–50)	18 (3–45)	14.5 (3–47)	<0.0001*
Baseline BCVA (log MAR)	1.06 ± 0.61	0.82 ± 0.54	1.28 ± 0.51	<0.0001*
1 month after baseline BCVA (log MAR)	1.0 ± 0.51	0.74 ± 0.55	1.07 ± 0.57	0.0005*
Final BCVA (log MAR)	1.0 ± 0.65	0.57 ± 0.59	0.84 ± 0.42	<0.0001*

SMH = submacular hemorrhage; tnAMD = typical neovascular age-related macular degeneration; PCV = polypoidal choroidal vasculopathy; RAM = retinal arterial microaneurysm.

*Statistically significant.

Within 1 month after baseline or intervention, vitreous hemorrhage occurred in 1 eye (3%) with tnAMD, 5 eyes (5%) with PCV, and 8 eyes (15%) with RAM. A macular hole was detected and treated in 2 eyes (2%) with PCV and 7 eyes (13%) with RAM, and the percentage was significantly higher in eyes with RAM (P = 0.0016). At 1 month, the mean CRT decreased significantly from baseline in all groups (all, P < 0.0001), from 628 to 327 μm in the tnAMD group, 550 to 275.5 μm in the PCV group, and 811 to 316 μm in the RAM group. The reduction in CRT from baseline to 1 month after treatment was greater in the RAM group than in the other groups but it did not reach statistical significance.

**Fig 2:**
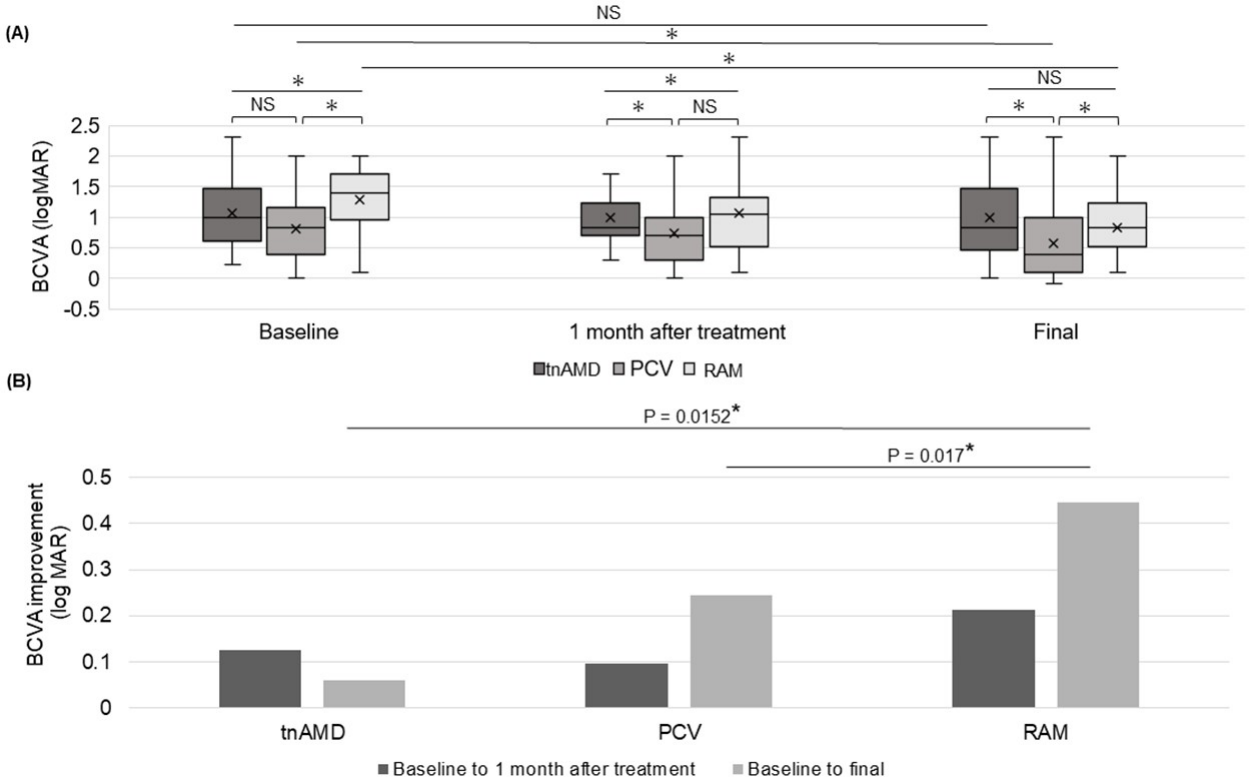
Comparison between the course of best-corrected visual acuity among the tnAMD, PCV and RAM groups. (A) Box plots of log MAR best-corrected visual acuity scores at baseline, 1 month, and at the final visit. (B) Improvement in the logMAR best-corrected visual acuity scores from baseline. BCVA: best-corrected visual acuity, logMAR: logarithm of minimum angle resolution, tnAMD: typical neovascular age-related macular degeneration, RAM: retinal arterial microaneurysm, PCV: polypoidal choroidal vasculopathy, NS: not significant. *P < 0.05. Statistical methods used were Wilcoxon’s rank test and paired *t*-test.

Table 3 and Fig 2A show the course of BCVA and Fig 2B shows the difference from the baseline in the BCVA at 1 month and the final visit. BCVA among the groups was significantly different at baseline, 1 month after baseline, and at the final visit (P < 0.0001, P = 0.0005, P < 0.0001, respectively). The final BCVA in the PCV group was significantly better than that in the tnAMD (P = 0.0007) and RAM (P = 0.0008) groups.

Although the BCVA at 1 month did not show a significant improvement compared with that at baseline in the tnAMD and PCV groups, the final BCVA was improved significantly in the PCV and RAM groups (P = 0.0009, P < 0.0001, respectively). The improvement in the final BCVA scores from baseline was significantly greater in the RAM group than in the tnAMD (P = 0.0152) and PCV (P = 0.017) groups (Fig 2B).

#### Factors affecting the final BCVA

A multivariate analysis was conducted to identify which factors affected the final BCVA, including age, baseline BCVA, thickness (T0 or T1+T3) and size (S1 or S2+S3) of SMH, layers involved in the hemorrhage (L0 or L1–4, L1 or L2–4, L1 or L2+3), baseline diseases (tnAMD, PCV, or RAM), duration from onset to intervention (AO or A1+A2), treatment or no treatment with PPV, and with or without anti-VEGF therapy after treatment (Table 4). Better BCVA at 1 month was significantly associated with better baseline BCVA (P < 0.0001) and SMH thickness of <500 μm (P = 0.0178). Better final BCVA was significantly associated with younger age (P = 0.0054), better baseline BCVA (P = 0.0021), and RAM subtype (P = 0.0446), and no tnAMD (P = 0.001).

**Table 4:** Factors associated with BCVA in eyes with SMH.

Preoperative parameters	BCVA at 1 month			Preoperative parameters	Final BCVA		
	Multiple linear regression analysis				Multiple linear regression analysis		
	Coefficient	95% CI	P		Coefficient	95% CI	P
Baseline BCVA	0.4	-0.17 to -0.02	<0.0001*	Age	0.01	0.004 to 0.02	0.0054*
Thickness of SMH (**T0**-T1&2)	-0.09	-0.17 to -0.02	0.0178*	Baseline BCVA	0.24	0.09 to -0.39	0.0021*
				RAM subtype	-0.15	-0.29 to -0.004	0.0446*
				No tnAMD	0.23	-0.09 to -0.37	0.001*

BCVA = best-corrected visual acuity; SMH = submacular hemorrhage; CI = confidence interval; RAM = retinal arterial microaneurysm; tnAMD = typical neovascular age-related macular degeneration.

*Statistically significant.

Group in bold font (T0) is the reference group.

In the PCV group, better final BCVA was significantly associated with younger age (P = 0.0372) and better baseline BCVA (P < 0.0001). In the RAM group, better final BCVA was significantly associated with younger age (P = 0.0002), SMH thickness of <500 μm (P = 0.0038), and no treatment with PPV (P = 0.0466).

## Discussion

In this retrospective multicenter study, we aimed to show the current clinical characteristics of, and treatment trends for, SMH in Japan.

In 1996, Berrocal et al. [15] evaluated 31 cases of SMH involving the fovea and found that 20 (64.5%) were associated with AMD and only 2 cases had RAM (6.5%). In 2015, Kim et al. [16] reported that the most common cause of SMH in South Korea was nAMD (52.9%), followed by PCV (37.3%), RAM (5.9%), and lacquer cracks (3.9%). Other surgical studies of SMH in different geographical areas showed that the cause of SMH was nAMD in 80%–90% of cases and RAM in 5%–10% of cases [1, 17, 18]. In the current study, the cause of SMH was nAMD in approximately 70% of cases (tnAMD 17% and PCV 50%) and RAM in approximately 30%, and this percentage of RAM was higher than in previous reports. This suggests that anti-VEGF therapy, which is currently widely used, can reduce bleeding in eyes with nAMD. Indeed, 40% of eyes in the tnAMD group and 30% in the PCV group had received previous anti-VEGF treatment.

Patients were significantly older and female sex was significantly more prevalent in the RAM group than in the nAMD group, and these background characteristics are important when estimating the etiology of SMH. Interestingly, the time from onset of SMH to the hospital visit was significantly longer in the tnAMD group, which might have been caused by a predetermined examination date for routine ani-VEGF treatment. An association between anticoagulant therapy and the development of SMH was reported in cases of nAMD [19–21]. In the current study, approximately 28% of patients were taking anticoagulant therapy, and this rate was not significantly different between the RAM and nAMD groups.

The OCT findings seemed helpful when differentiating between nAMD and RAM in eyes with SMH. In agreement with previous reports, the current study showed that eyes with RAM were most likely to have hemorrhage involving more than two layers including the sub-hyaloidal/sub-ILM space and no sub-RPE hemorrhage, whereas eyes with nAMD were more likely to have subretinal and sub-RPE hemorrhage and no preretinal hemorrhage [1, 19].

Nearly 60% of eyes with nAMD had a hemorrhage thickness of <500 μm, whereas nearly 60% of eyes with RAM had a hemorrhage thickness of ≥500 μm. The CRT was significantly thicker in patients with RAM compared with nAMD. In contrast, the size of SMH was significantly larger in patients with nAMD than with RAM. Thus, patients with RAM tended to have a thicker hemorrhage, and patients with nAMD tended to have a larger hemorrhage. These findings seem reasonable because a large lesion size and sub-RPE location of choroidal neovascularization or PCV may cause a wider spread of subretinal hemorrhage, and bleeding from RAM surrounding the retinal layers may spread transversely with high arterial blood pressure.

Although various treatments for SMH have been shown to be effective, [3] no consensus has been reached on the optimal treatment for SMH; thus, the choice of treatment depends on the patient and doctor. In the current study, even after SMH, anti-VEGF therapy with and without expansile gas injection was conducted for half of the eyes in the tnAMD and PCV groups. Anti-VEGF therapy is currently the mainstream treatment for nAMD. Studies have shown that thinner or smaller SMH can be treated with anti-VEGF monotherapy, [22, 23] and our study confirmed that the intravitreal injection of anti-VEGF with or without other combined therapy is now an important treatment option for significant SMH.

For SMH associated with RAM, 84% of cases were treated with PPV and pneumatic displacement. Preretinal hemorrhage, especially sub-ILM hemorrhage, is often seen in association with SMH in eyes with RAM, [24] and PPV can be primarily chosen for its removal. In contrast, PPV was chosen for about a quarter of eyes with nAMD, and it was frequently combined with subretinal tPA injection. Considering the necessity of further anti-VEGF treatment, PPV has less priority because of the short half-life of the intravitreal anti-VEGF drug in vitrectomized eyes [25]. However, a high volume of gas can be injected intravitreally into vitrectomized eyes, which enables further SMH displacement for a longer period than in non-vitrectomized eyes [26]. A previous study showed that the visual outcomes were similar between eyes treated by the subretinal injection of tPA or intravitreal tPA with pneumatic displacement, but the final scar area was significantly smaller in patients treated with subretinal tPA [27]. Therefore, when PPV is chosen for eyes with nAMD, subretinal tPA injections might be reasonable for the displacement of large SMH and sub-RPE hemorrhage [28]. Statistically, our study indicated that better baseline BCVA, diagnosis of RAM, no diagnosis of tnAMD, and shorter duration of SMH were factors for choosing PPV.

A previous study showed that the visual prognosis of nAMD-related SMH is worse than that of RAM [1]. The current study revealed that the BCVA of PCV was significantly better than that in the other groups throughout the study period, whereas the BCVA of tnAMD did not improve significantly from baseline to final visit. Many studies have shown that the visual prognosis is better in PCV than in tnAMD [29] although PCV is well known for its hemorrhagic nature [5]. In this study, multivariate analysis showed that better final BCVA was significantly associated with younger age, better baseline BCVA, and not diagnosed as tnAMD; these factors are characteristic of PCV.

A diagnosis of RAM was another factor indicating a better final BCVA. Because RAMs are not located at the fovea, a better VA might be achieved when SMH is successfully removed from the subfoveal region. The current data revealed that the RAM group also showed more significant visual improvement between baseline and the final visit than the other groups and we had expected PPV might explain a greater improvement in BCVA in this group because PPV had been chosen in most cases. However, unexpectedly, the analysis showed that PPV was a predicting factor for worse BCVA in the RAM group. Intraretinal hemorrhage is a key factor for poor visual prognosis in eyes with RAM, [30] and eyes with hemorrhage in both preretinal and subretinal spaces that could be primarily treated with PPV have more chances of intraretinal hemorrhage including the fovea. The higher incidence of macular hole formation might be another reason for the worse BCVA associated with PPV.

This study had several limitations. First, this was a retrospective study, not a randomized study; therefore, the cases included in this study may not reflect real clinical cases of SMH. Patients who preferred observation without intervention might not have been referred to the facilities in J-CREST. In addition, the treatment strategies depended on the handling doctor. Second, not all cases were examined using swept source-OCT although conventional spectral domain-OCT is known to have a limited capability for the evaluation of retinal layers beneath a massive SMH. However, the FLATCAPS classification used in this study did not require an exact thickness or layers, so we think this did not affect the results. The large number of SMH cases in this study allowed us to determine the current thinking related to the characteristics and treatment strategies for SMH in Japan.

In conclusion, this retrospective multicenter survey in Japan identified differences in the patient characteristics, ophthalmic findings, selected treatments, and visual prognoses among SMH with tnAMD, PCV and RAM. BCVA improvement was more significant in the RAM group than in the other groups because more cases received PPV. Final BCVA was better in PCV, RAM, and tnAMD, in that order, probably because cases with PCV were younger and had better baseline BCVA than those with other etiologies.

## Supporting information

S1 File Original data used in this article are included. (XLSX)
